# Precision Dosing Priority Criteria: Drug, Disease, and Patient Population Variables

**DOI:** 10.3389/fphar.2020.00420

**Published:** 2020-04-22

**Authors:** Rachel J. Tyson, Christine C. Park, J. Robert Powell, J. Herbert Patterson, Daniel Weiner, Paul B. Watkins, Daniel Gonzalez

**Affiliations:** ^1^ Division of Pharmacotherapy and Experimental Therapeutics, UNC Eshelman School of Pharmacy, The University of North Carolina at Chapel Hill, Chapel Hill, NC, United States; ^2^ Institute for Drug Safety Sciences, The University of North Carolina at Chapel Hill, Chapel Hill, NC, United States

**Keywords:** precision dosing, individualized dosing, therapeutic index, pharmacokinetics/pharmacodynamics, biomarkers, disease states, pharmacoeconomics, drug development

## Abstract

The administered dose of a drug modulates whether patients will experience optimal effectiveness, toxicity including death, or no effect at all. Dosing is particularly important for diseases and/or drugs where the drug can decrease severe morbidity or prolong life. Likewise, dosing is important where the drug can cause death or severe morbidity. Since we believe there are many examples where more precise dosing could benefit patients, it is worthwhile to consider how to prioritize drug–disease targets. One key consideration is the quality of information available from which more precise dosing recommendations can be constructed. When a new more precise dosing scheme is created and differs significantly from the approved label, it is important to consider the level of proof necessary to either change the label and/or change clinical practice. The cost and effort needed to provide this proof should also be considered in prioritizing drug–disease precision dosing targets. Although precision dosing is being promoted and has great promise, it is underutilized in many drugs and disease states. Therefore, we believe it is important to consider how more precise dosing is going to be delivered to high priority patients in a timely manner. If better dosing schemes do not change clinical practice resulting in better patient outcomes, then what is the use? This review paper discusses variables to consider when prioritizing precision dosing candidates while highlighting key examples of precision dosing that have been successfully used to improve patient care.

## Introduction

Precision dosing (also referred to as individualized dosing) utilizes drug attributes [*e.g.,* narrow therapeutic index (NTI), pharmacokinetic/pharmacodynamic (PK/PD) variability], disease state characteristics (*e.g.,* extent of morbidity and/or mortality) as well as patient-specific factors (*e.g.,* organ function, gene variants), to optimize drug therapy. Drugs play an essential role in human health, with the goal of choosing the right drug and dose for the right patient remaining an ever-present challenge for clinicians. Historically, pharmacies and pharmacists used compounding as a common approach to individualize prescriptions to provide therapy in different formulations and doses not widely available. Individualized therapies in the form of compounded products significantly diminished as mass manufacturing of drug products began in the middle of the 20^th^ century (Lesko and Schmidt, 2012). The 20^th^ century also marked the beginning of the modern era of individualized dosing with the isolation and purification of insulin to treat high blood sugar (Bliss, 1982). Today, individualized drug dosing is underutilized, as modern medicine routinely follows standard dosing established by randomized controlled trials, which are viewed as the gold standard for evidence-based medicine. There is an opportunity to greatly improve patient care with precision dosing as the health care system continues to evolve.

Drugs are not benign in that nearly all have adverse effect profiles with varying degrees in response rates even when taken as studied and prescribed. Therefore, it is important that all drugs, particularly those used to treat serious illnesses or those in which the exposure window between efficacy and toxicity is narrow, are well managed. Clinicians regularly adhere to standard recommendations for initial dosing which may not be ideal or safe for all patients, particularly if the drug has not been studied in patient populations with different dose–exposure and/or exposure–risk relationships. Subsequent titration of the dose for efficacy or safety may be implemented but such a strategy is inefficient and delays the benefits received from therapy. Imprecise drug dosing in certain subpopulations as a result of standard, fixed dosing methods or gaps in knowledge carries increased risks for potentiating adverse events due to supratherapeutic or subtherapeutic concentrations (Watanabe et al., 2018). Suboptimal drug exposure can then lead to poor efficacy and safety outcomes ranging from minor to severe, depending on the dose and patient to which the drug was administered. Tailoring drug therapy with consideration to the drug, disease state, and patient enhances the probability to achieve efficacy and minimize adverse effects.

**Figure 1:**
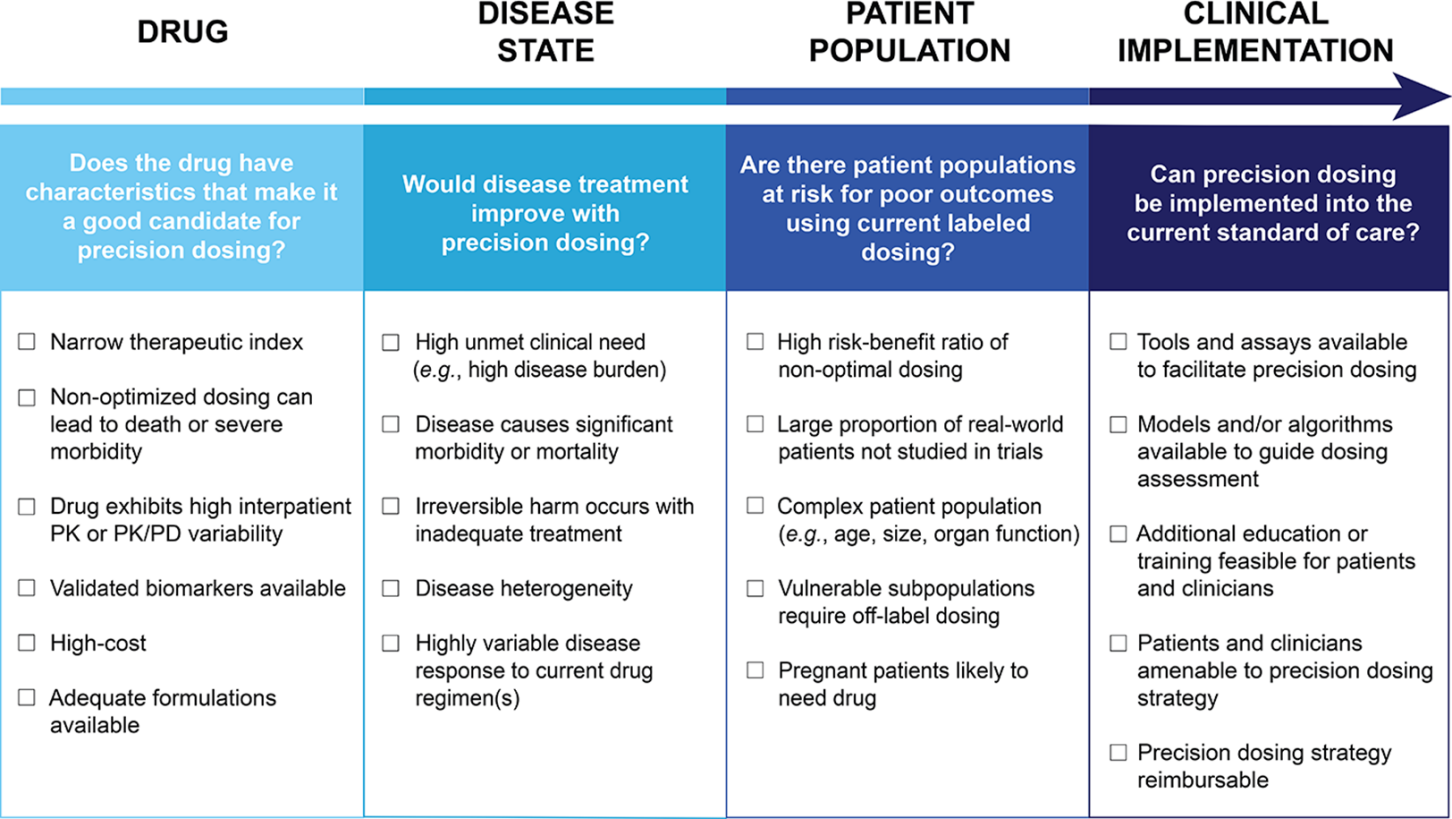
Assessment of candidacy for precision dosing. The considerations to guide the assessment of candidates for precision dosing are outlined. Drug, disease state, patient population, and clinical implementation are all areas that could influence decisions on precision dosing. These categories can be used to help think through both clinical and logistical concerns related to integrating the precision dosing of a drug into practice. PK, pharmacokinetic; PK/PD, pharmacokinetic/pharmacodynamic.

Though there are some drugs for which the benefits of precision dosing have been established (Gonzalez et al., 2017), there is no widely accepted approach to determine which drugs should be prioritized for precision dosing, nor which drug and disease criteria should be considered. Therefore, we propose that the need for precision dosing can be informed by the following drug, disease state, and patient population related variables: A drug’s therapeutic index, the extent of PK/PD variability in patients, availability of biomarkers to facilitate individualized dosing, disease state considerations, pharmacoeconomics, and disparity between phase II/III trial patients and real-world patients. These factors can be assessed to determine if a drug should or should not be a precision dosing candidate. **Figure 1** outlines key drug, disease state, patient population, and clinical implementation considerations that can be used to guide the assessment of precision dosing candidates. For some drugs, the decision will be clear cut, while for others, each of the factors will need to be carefully weighed. The basic question is: Are there likely to be patients who will receive the labeled dosage regimen who are either unlikely to experience efficacy or likely to experience toxicity because of their characteristics? This should be an important question in all instances, but it is particularly important when the anticipated outcome is serious.

Precision dosing has the potential to elevate the overall quality of drug therapy to provide improved care for patients in whom standard labeled dosages are suboptimal. Current Food and Drug Administration (FDA) regulations generally only require a drug to be statistically significantly better versus placebo or noninferior as compared to the current treatment standard. This does not guarantee that the drug is effective in a majority of patients studied in clinical trials, such as in difficult to treat cancers including diffuse intrinsic pontine glioma (DIPG) and unresectable meningioma, where the response rate to treatments can be extremely low (Ji et al., 2015; Fleischhack et al., 2019). Additionally, unless the phase III to real-world patient gap has been defined, it is not known if populations studied in phase III trials are an accurate representation of the entire treatable population. Standard, fixed dosing regimens approved by the FDA are thus an impediment to principles of precision dosing. Individualizing doses with consideration to drug, disease state, and patient-specific factors supports the shift toward value-based patient-care models to better outcomes in more diverse patient populations. As such, it will be integral to prioritize candidates for precision dosing to direct financial, time, and health care resources. This review paper provides evaluations of factors and key examples to consider when determining the candidacy of drugs for precision dosing. For some drugs, there lacks sufficient information available to guide precision dosing decisions. Therefore, the paper also discusses ways of adapting the drug development process to inform and facilitate precision dosing efforts in the future.

## Therapeutic Index

An important variable to take into consideration when determining priority drug candidates for precision dosing is the therapeutic index. The therapeutic index depends on various drug-specific factors and describes the ratio between a drug’s maximum tolerated dose and lowest effective dose (Levy, 1998). Drugs with a large therapeutic index (exceeding a value of 10) can be dosed in most patients without causing adverse events or therapeutic failure (Tamargo et al., 2015). Drugs with a NTI must be dosed more carefully and have a smaller drug exposure window between toxic and therapeutic effects. These are also called “critical-dose drugs” and often require therapeutic drug monitoring (TDM) and dose individualization based on patient-specific characteristics (Pater, 2004). Values of ≤2 (Bialer et al., 1998; Greenberg et al., 2016; Ericson et al., 2017) or ≤3 (Li et al., 2018) have been considered therapeutic index cut off points for NTI drugs. Examples of drugs that have been specified as NTI by regulatory agencies include anticoagulants (*e.g.,* warfarin), antiarrhythmics (*e.g.,* digoxin, flecainide), antiepileptics (*e.g.,* phenytoin, carbamazepine), hormones (*e.g.,* levothyroxine, ethinyl estradiol), and immunosuppressants (*e.g.,* tacrolimus, sirolimus, cyclosporine) (Yu, 2011). A review performed by Li et al. contains a more comprehensive list of drugs that could be considered NTI that also includes antimicrobials, oncology drugs, and opioids (Li et al., 2018). The majority of these potential NTI drugs are not classified as such by the FDA, which is partially due to the difficulty of characterizing the therapeutic index.

There is a lack of literature describing how therapeutic index should best be determined, but several approaches are possible depending on available information (Muller and Milton, 2012). During drug development, the therapeutic index can be estimated using relevant safety and efficacy data generated in *in vitro* and *in vivo* studies. Muller and Milton suggest calculating therapeutic index with the use of drug exposure at steady state. Maximum concentration (C_max_), minimum concentration (C_min_), and area under the concentration versus time curve (AUC) are important parameters that can be used to assess exposure and thus therapeutic index (Muller and Milton, 2012; Suzuki et al., 2012). The therapeutic index is unknown for many drugs, even after approval and widespread use (Ku et al., 2016). For approved drugs, the therapeutic index can be estimated with pharmacokinetic (PK) modeling using routine TDM and electronic health record (EHR) data. This method performed by Ku and colleagues accurately determined the therapeutic index of phenytoin with data that had been collected from patients during standard care (Ku et al., 2016). Systematic review of published data has also been used to estimate the therapeutic index in approved drugs (Greenberg et al., 2016; Ericson et al., 2017). In these studies, safety, efficacy, and therapeutic monitoring data were gathered from published trials and successfully used to estimate the therapeutic index of antiepileptics (phenytoin, phenobarbital, and valproate) (Greenberg et al., 2016) and an immunosuppressant (cyclosporine) (Ericson et al., 2017). However, because patients and physicians are reluctant to collect data that requires frequent monitoring unless it is necessary, many drugs may not have enough TDM data to be able to use this type of method. Additionally, the validation and comparison of different commercial assays used to measure drug concentrations can lead to data misinterpretation when multiple studies are assessed in meta-analyses. Structured registries such as quality registries implemented in collaboration with health authorities can aid in the collection of clinical data in a relevant number of patients thus allowing more comprehensive interpretation of data (Bhatt et al., 2015). Despite limitations associated with these approaches, they represent low-risk and low-cost methods of classifying a drug’s therapeutic index (for drugs with a wealth of TDM and other information) which is important in the regulation of the development of generic drugs.

Generic drugs must perform similarly to their brand-name counterparts, especially for NTI drugs. Biosimilars, while not classified as generic, are highly similar to the reference product (biologic agent) and may provide a comparable benefit–risk profile. When two drug products are bioequivalent, it is expected that they can be used interchangeably to produce the same therapeutic effect (Chow, 2014). However, bioequivalence does not always equal therapeutic equivalence, especially in NTI drugs (Burns, 1999). Generics have different excipients that may interfere with some assays and concerns have arisen with the adequacy of generic substitution of drugs such as warfarin (Dentali et al., 2011), levothyroxine (Carswell et al., 2013), phenytoin (Kinikar et al., 2012), and tacrolimus (Sommers et al., 2013). Historically, clinicians have favored more strict bioequivalence guidelines for NTI drugs such as these (Banahan and Kolassa, 1997). In order to address these concerns and improve the generic drug approval process, bioequivalence standards for NTI drugs were made more stringent in 2010. While the FDA bioequivalence standard allows for the confidence limits on the ratio of formulation means for AUC and C_max_ to differ by as much as 20% for all non-highly variable drugs, NTI drugs are now limited to 11% variability in these parameters (Jiang et al., 2015). Bioequivalence studies must be performed using these criteria in order to prove that a NTI generic drug has the same clinical effects as a reference drug. Improper application of stricter bioequivalence criteria to non-NTI drugs would result in bioequivalent generic drugs not being approved. Conversely, if a NTI drug was misclassified as non-NTI, standard bioequivalence criteria would be applied, thus potentially leading to the approval of a generic drug that has a more variable dose–exposure relationship than anticipated. Therefore, timely and correct identification of NTI drugs is an important foundation for bioequivalence testing. For example, the NTI designation could occur at New Drug Application (NDA) approval. Generic drugs play a major role in health care and both accurate bioequivalence and therapeutic index information are necessary for optimal dosing.

Precision dosing is likely not necessary for drugs with a therapeutic index over 10 (unless major cost savings could be achieved through more efficient use of a drug product) but may greatly benefit drugs with narrow therapeutic indices. Drugs that fall into the NTI category (therapeutic index of 2–3 or below) often require close monitoring and dose titration that would not be necessary for non-NTI drugs. A study conducted in Norwegian hospitals found that drug-related problems were more likely to be associated with NTI drugs as opposed to non-NTI drugs, results which were driven by drug interactions, the need for increased patient monitoring, and non-optimal dose assignment (Blix et al., 2010). When NTI drugs are dosed incorrectly, serious consequences can occur. For example, a dose of digoxin that is too high for a patient can cause severe toxicity and even death. Only two and a half times a normal dosage of digoxin can be fatal in 50% of patients (Burns, 1999; Hu et al., 2018). NTI drugs are especially dangerous in patients that are elderly, have multiple illnesses, or are receiving multiple drugs (Burns, 1999). Patients on numerous drugs are more likely to experience drug interactions that can lead to drug concentrations that are too high or too low. Even drug-food interactions can have a major impact on the amount of drug in a patient’s body. In vulnerable patients, sometimes NTI drugs cannot be avoided and it is essential that the most optimal regimens are chosen.

For drugs whose therapeutic index falls between 3 and 10, the utility of precision dosing is less clear, but there is still significant opportunity for benefit. To determine suitability of precision dosing in these drugs, it is particularly important that factors other than therapeutic index alone are considered. Other variables that should be considered include biomarker availability, disease state, pharmacoeconomics, the information gap between real-world patients and clinical trial patients, and PK/PD variability.

## PK/PD Variability

Inter-individual PK/PD variability can affect the need to implement precision dosing across diverse patient populations. Dose–exposure and exposure–response relationships may vary despite administration of the same dose due to inter-individual variations in patient characteristics such as weight, organ function, age, and genetic variance. For example, at the upper extremes of body weight, physiologic changes (*e.g.,* increased ratio of adipose tissue to lean body mass and increased cardiac output leading to more blood flow to the liver and kidneys) result in fluctuations in the volume of distribution (V_d_) and clearance of drugs, complicating dosing recommendations (Smit et al., 2018). Traditional dosing for oncology drugs utilizes body surface area (BSA)-based dosing regimens to address variability in drug exposure (Gurney, 1996), though large inter-individual variability still exists (Sawyer and Ratain, 2001). A review by Baker et al. demonstrated that BSA-based dosing as a method to reduce inter-individual variability was only useful in a small number of chemotherapy agents (Baker et al., 2002). Organ function can also introduce significant variability in PK/PD as impairment of liver and kidney function can result in metabolism changes and reduced clearance, often leading to unique dosing needs for patients. This is evidenced in the geriatric population which experiences reduced renal and hepatic clearance, decreased microbiota diversity (further reducing drug metabolism and clearance) (Enright et al., 2016; Ticinesi et al., 2017; Mangiola et al., 2018), increased sensitivity to certain NTI drug classes [*e.g.,* anticoagulants and central nervous system (CNS) depressants], and increased frailty as compared to younger counterparts (Mangoni and Jackson, 2004). Individualized dosing considering these factors in the vulnerable geriatric patient population can mitigate the risks for toxicity and poor outcomes. Genetic polymorphisms in drug metabolizing enzymes can also influence the dose–exposure relationship of drugs, particularly those affecting the cytochrome P450 (CYP450) system which has been reported to metabolize 70%–90% of small molecule drugs used today (Furge and Guengerich, 2006; Lynch and Price, 2007; Zanger and Schwab, 2013; Parker et al., 2016). A commonly used antiplatelet agent, clopidogrel, is unable to be transformed *via* CYP2C19 to its active metabolite by patients classified as poor metabolizers, thereby decreasing the effectiveness of the drug (Kim et al., 2008; Jang et al., 2012; Bristol-Meyers Squibb, 2019). The likelihood of adverse outcomes can be reduced or possibly avoided by identifying genetic polymorphisms likely to reflect poor responders and adjusting the drug’s dosage accordingly. Inter-individual differences such as genetic variations as well as weight, organ function, and age can significantly alter drug PK/PD, and therefore influence the need for a drug to be dosed differently. Significant inter-individual variability for certain drugs may also represent a greater need for precision dosing, though intrinsic drug characteristics also cause variability in PK/PD between patients and must be considered as well.

Drug characteristics can impact the extent of PK/PD variability and need for precision dosing. For drugs that have high intrinsic variability, intra-individual variability can also factor into dose assessment. For example, tacrolimus exhibits variable bioavailability, multiple drug interactions, and unclear exposure–response correlations (such as the correlation between subtherapeutic concentrations and risk of organ rejection). Bioavailability refers to the rate and extent of absorption and is an important source for both inter- and intra-individual plasma concentration variation. It can be influenced by both drug route and formulation, though drugs also exhibit variations in bioavailability within the same formulation. The bioavailability of tacrolimus ranges from 4% to 89% after oral administration, presenting challenges in achieving and maintaining therapeutic concentrations (Wallemacq and Verbeeck, 2001; Gueta et al., 2018). Therefore, therapeutic monitoring is required to ensure patients receive optimal exposure to the drug. Supratherapeutic tacrolimus concentrations may increase the risk of nephrotoxicity while subtherapeutic concentrations may increase the risk of antibody-mediated organ rejection over time (Sikma et al., 2015; Mendoza Rojas et al., 2019). Drug concentrations may also be altered by drug-induced PK changes. Drug disposition is often impacted by drug metabolizing enzymes and transporters such as CYP450, glucuronidases, P-glycoprotein (P-gp), and organic-anion-transporting polypeptide 1B1 (OATP1B1) to varying extents. Disposition *via* these drug metabolizing pathways or transporters can be affected by inhibitor/inducer properties of co-administered drugs. For example, as a substrate of both CYP3A and P-gp, tacrolimus concentrations will increase or decrease with concomitant administration of drugs that inhibit or induce these pathways, respectively (Staatz and Tett, 2004). Drugs to be administered concomitantly with an agent that affects its metabolism will benefit from precision dosing recommendations considering the impact of these interactions on systemic exposure and efficacy. While bioavailability and drug interactions highlight variability in dose–exposure relationships, variability may also be evidenced in exposure–response relationships. The pharmacodynamic (PD) variations in tacrolimus dosing are less elucidated than the PK relationships seen with bioavailability and drug interactions. It may be expected that reduced tacrolimus exposure will increase the incidence of rejection while increased exposures potentiate the risk for toxicity and over-immunosuppression (Christians et al., 2002); however, changes in blood concentrations are not always directly related to responses in efficacy or toxicity. Though several studies have indicated an increased risk of nephrotoxicity with elevated tacrolimus trough concentrations (Bäckman et al., 1994; Kershner and Fitzsimmons, 1996), there are conflicting reports as to whether low tacrolimus concentrations can be related to organ rejection (Staatz and Tett, 2004). Precision dosing may not be appropriate for all drugs or indications, since in some instances the relationship between drug exposure and drug response may not be known or well understood. However, precision dosing may improve current reactive dosing strategies for some drugs by evaluating better predictors of dose response and exposure variability on clinically meaningful outcomes.

Within subject variabilities such as inter-occasion and intra-individual variability can be unpredictable and present challenges for precision dosing. Inter-occasion variability (IOV) is a function of time defined as differences occurring within the same patient at separate time points (Holford and Buclin, 2012). The impact of IOV may be characterized but requires sufficient data across variable time points to inform dosing recommendations. IOV can be estimated in population PK models though there are challenges in precision with high magnitudes of IOV (Abrantes et al., 2019). Abrantes et al. evaluated five approaches to address high IOV and concluded that methods excluding the impact of IOV for individualized dosing were most accurate (though including IOV estimations for empiric Bayesian estimates was found to be most accurate and precise) (Abrantes et al., 2019). For situations in which the IOV is expected to be greater than inter-individual variability, IOV should be excluded from dosing recommendations due to the lack of predictive ability. Notably, patient compliance should also be assessed at regular visits as non-adherence creates further variability in drug concentration monitoring between visits. Investigation of reasons for non-adherence may also provide valuable clinical knowledge as discontinuation due to patient perceived improvement as opposed to intolerance to therapy are meaningful differences. While IOV explains variations within the same patient on different occasions, intra-individual variability describes discrepancies within the same patient at the same visit. Intra-individual variability is a component of random unexplained variability and residual error as evidenced by assay errors, uncertain dosing times and imperfect models (Abrantes et al., 2019). These random sources of variability cannot be explained and therefore are an obstacle to model-informed precision dosing. Precision dosing methods are most valuable when variability can be readily estimated, including inter-individual variability, but are challenged with large magnitudes of unpredictable, random IOV and large intra-individual variability.

Model-informed precision dosing can be implemented to account for PK/PD variability to determine appropriate doses based on sources of inter-individual variability. Nonlinear mixed effects modeling (*e.g.,* population modeling), Bayesian forecasting, and physiologically-based PK/PD (PBPK/PD) modeling are tools that can be used to account for sources of PK/PD variability (Gonzalez et al., 2017). Population PK models incorporate patient-specific covariates and provide *a priori* dosing recommendations based on predicted PK parameter estimates (Vinks, 2002). Population PK models can also be applied to TDM-based *a posteriori* dosing with the application of Bayesian estimations to create an individualized patient-specific model (Gonzalez et al., 2017). Feedback from TDM allows refinement of PK parameter estimations to allow progressive updating of parameters within the model to enable more accurate predictions for subsequent dosing (Jelliffe et al., 1993). Bayesian guided dosing has shown excellent predictive performance in various clinical studies though estimation methods heavily rely on the availability and accuracy of population PK models (de Jonge et al., 2004; Mar Fernández de Gatta et al., 2009). For patients lacking sufficient data to inform population PK models, PBPK/PD models may be useful as dose predictions are mechanistic-based and derived from known impacts of organ/tissue function on PK/PD, though significant understanding of the metabolism and *in vitro* preclinical data is required for accuracy (Polasek et al., 2018). PBPK/PD modeling offers the advantage of drawing from human physiology, drug physicochemical properties, and enzyme/protein variability in patients to individualize dosing and has been successfully used in estimating drug exposure (Gonzalez et al., 2017; Polasek et al., 2019). Physiologically-based models typically focus on estimating dose–exposure but increased investigation on PD effects will better incorporate exposure–response effects for PBPK/PD modeling.

When a drug is used across diverse patient populations, patient-specific factors, drug characteristics, and disease-specific considerations (particularly when a drug is used for different indications) can contribute to variable PK/PD. Precision dosing can be useful to address these sources of variability and adjust dosing recommendations accordingly. Model-informed dosing recommendations utilizing covariates for dosing predictions may address inter-individual variability, but such approaches are challenged by IOV and intra-individual variability which may remain unexplained. The determination to implement precision dosing should not be solely based on PK/PD variability as such variability is only significant if concentration fluctuations occur outside the range associated with efficacy and safety. Considering additional factors, such as therapeutic index, in conjunction with PK/PD variability can help assess the need for precision dosing.

## Biomarkers

A biological marker (biomarker) is understood to be “a defined characteristic that is measured as an indicator of normal biological processes, pathogenic processes, or responses to an exposure or intervention, including therapeutic interventions” (Robb et al., 2016). These measures can be used to characterize the body’s response to a therapeutic intervention and represent a valuable yet underutilized avenue for precision dosing. Examples of biomarkers used to guide therapy include hemoglobin, international normalized ratio (INR), pharmacogenomic results, and drug concentrations (Jones, 2011; U.S. Food and Drug Administration, 2015; Gonzalez et al., 2017; Meaney et al., 2019). For a biomarker to be routinely used in health care it needs strong analytical validity as well as clinical validity and utility. Analytical validity refers to the reliability of the test associated with the biomarker, clinical validity is how well the test measures the clinical feature of interest, and clinical utility describes the applicability of the biomarker to clinical use (Kraus, 2018). A robust biomarker must be measurable *via* an accurate and reproducible assay, relevant as a guide to clinical decision making, and informative of decisions that lead to favorable patient outcomes. The ideal biomarker would also be straightforward to interpret and easily incorporated into clinical care. Biomarker tests which are readily available, low cost or reimbursable, and quickly performed are more likely to be used frequently (Ciardiello et al., 2016). Several biomarkers are commonly used for facilitating dose individualization and are associated with improved patient outcomes. For instance, the INR is used as a monitoring biomarker to adjust warfarin dosing. The recommended goal INR range for most warfarin patients is between 2.0 and 3.0 (Holbrook et al., 2012) and increased time in this range is correlated with improved patient outcomes (Baker et al., 2009). INRs above or below the therapeutic range are associated with increased risk of bleeding events or strokes, respectively (Reynolds et al., 2004). Additionally, serum immunoglobulin E (IgE) is used as a biomarker to optimize dosing of omalizumab, a monoclonal antibody (mAb) used to treat severe allergic asthma (Lowe et al., 2015). Both baseline IgE concentrations as well as body weight are used to determine dose and frequency of omalizumab. More research is needed to identify additional biomarkers that may be used to individualize dosing.

Biomarkers are an important component of the drug development process and can play a role in patient selection for clinical trials, toxicity monitoring, and guidance of dose selection (Drucker and Krapfenbauer, 2014). In order to properly characterize biomarkers that have potential utility in precision dosing, certain data needs to be collected during the drug development process. Clinical trials should require genetic information from participants when there is evidence of genomic influence on drug response. An analysis of ClinicalTrials.gov found that less than 1% of registered clinical trials included pharmacogenomics outcomes (Burt and Dhillon, 2013) despite potential genetic predictors of efficacy that could influence clinical decision making. Although the majority of drug developers attempt to identify biomarkers predicting response or safety, this data is rarely published. Additionally, drug concentration measurements are a valuable source of information which few phase III trials collect comprehensively. PK samples should be collected during phase III trials where possible and used in a pooled analysis that includes data from phase I and II studies that used more intensive sampling. Biomarker data once available can then be used in further research to investigate the connection between biomarkers and drug response. PK/PD models can incorporate biomarkers in order to monitor adverse events early, predict clinical response, and predict concentrations of a drug that will produce an effect. Warfarin (Hamberg et al., 2010), sitagliptin (Kim et al., 2013), cyclooxygenase-2 inhibitors (Huntjens et al., 2005), and sunitinib (Hansson et al., 2013) have associated biomarkers that have been incorporated into PK/PD models. Hansson et al. developed PK/PD models of sunitinib using angiogenic biomarker candidates including vascular endothelial growth factor (VEGF), soluble VEGF receptor (sVEGFR)-2,-3, and soluble stem cell factor (sKIT). These biomarker candidates were tested in the models to predict adverse events including myelosuppression, fatigue, and hand-foot syndrome. sVEGFR-3 was found to be the most effective predictor of these adverse events. The study concluded that early monitoring of sVEGF-3 to identify patients at highest risk for toxicity could inform dose individualization of sunitinib and ultimately improve survival (Hansson et al., 2013). Given the potential impact of biomarkers on patient outcomes, there is increasing interest in identifying and collecting data on relevant biomarkers during studies. The FDA encourages the integration of biomarkers into drug development and has developed the “Biomarkers, EndpointS, and other Tools” (BEST) resource to improve efficiency of biomarker development (Califf, 2018). Continued improvement of biomarker study in clinical trials is needed to facilitate further dose optimization strategies.

While biomarkers have the potential to be a key component of precision dosing, translation of biomarker information to clinical practice is lacking. Thousands of biomarkers have been discovered, but only around 100 are used routinely in the clinic (Poste, 2011). Validation of biomarkers for use in the clinic presents both logistical and regulatory challenges and requires large-scale, expensive studies (Poste, 2011). Other major obstacles can include expense, laboratory errors, variability in biomarker results, and misinterpretation of biomarker results (Mayeux, 2004). To be useful for precision dosing, a good biomarker must be able to provide insight on dose adjustment despite interfering intrinsic and extrinsic factors. Diurnal and disease mediated fluctuations, timing of biomarker collection, and drug effects on a biomarker are all factors that can change the results of a biomarker test. For example, it is known that INR can be impacted by many variables including dietary vitamin K intake, liver disease, and recent surgery (White, 2010). Any biomarker fluctuation needs to be characterized and well understood in order to use a biomarker for precision dosing. Another practical consideration related to the use of a precision dosing biomarker is prescriber training and knowledge. Clinicians commonly use information such as patient age, body size, and comorbidities to guide dosing, but may not feel suitably trained to make clinical decisions using biomarkers. Ideally, sufficient guidance would be provided by the biomarker’s developer including information on the biomarker test, possible results, and recommended therapy modification based on results. Biomarkers introduce additional complexity to the prescribing process and most prescribing information does not include comprehensive information relevant to subpopulations (such as biomarker data).

Despite challenges associated with biomarker use and implementation, there remains opportunity for biomarkers to serve as a valuable source of information to help clinicians select the best dose for each patient. Biomarker analyses facilitate better understanding of drug disposition as well as drug response and can identify subgroups of patients that may benefit from individualized dosing. Ultimately, improvements that a biomarker can make in drug safety and efficacy must outweigh cost and any associated inconvenience. The benefit–risk profile of a drug with and without the use of a precision dosing biomarker should be assessed in order to determine if there is an advantage to using the biomarker. If a biomarker leads to a favorable benefit–risk profile and can be used to individualize dosing, it should be incorporated into clinical practice. Drugs that are accompanied by actionable biomarkers that improve outcomes through dose optimization are more likely to be selected by future payers and prescribers.

## Disease State Considerations

Disease state is one of the most important factors to consider when determining if a drug should be prioritized for precision dosing. The integration of precision dosing is likely to be most helpful in areas of high unmet medical need (Darwich et al., 2017), which include infectious disease, hematology, immunology/transplantation, oncology, neurology, and other therapeutic areas noted by Scavone and colleagues (Scavone et al., 2019). Disease related morbidity, mortality, and progression can be quite variable and can greatly impact the need for drug dosing individualization. If precision dosing were to result in substantial mortality reductions for a disease/drug combination, that alone would likely outweigh any factors that indicated that a drug would otherwise not be a good candidate for precise dosing. Similarly, the case for precision dosing would be compelling if significant improvements in morbidity were possible, despite potential trade-offs. Precision dosing comes at a cost, both in drug development and clinical practice, whether it be for researching biomarkers, training clinicians, or developing software. Because of this, there must be strong rationale for how precision dosing for a drug will be advantageous to the patient, providers, health system, and payers. However, it is also important to note that the cost of several samples during a clinical trial (which can provide unique insight into the need for dosing individualization) is comparatively low compared to the trial cost overall. Ultimately, precision dosing is likely to be worth the costs in cases where it can significantly improve morbidity and mortality or fill a gap in medical need.

Certain diseases and drugs are responsible for a high burden of morbidity and mortality, and therefore may significantly benefit from precision dosing. Infectious disease is a major cause of morbidity and mortality worldwide (Christensen et al., 2009), with infections such as sepsis and pneumonia frequently causing patients to be hospitalized (Kennedy et al., 2019). Antibiotic classes commonly used to treat serious infections include aminoglycosides, beta-lactams, fluoroquinolones, and glycopeptides (Nemeth et al., 2015). TDM is already successfully used in aminoglycoside and vancomycin dosing, but may benefit other classes such as beta-lactams and fluoroquinolones that have variable PK and problems with subtherapeutic dosing (Roberts et al., 2012). Cancer is also among the leading causes of death worldwide, and is becoming more common as the population ages (Collins and Varmus, 2015). Many cancers are treated with toxic drugs that include traditional intravenous chemotherapy as well as newer oral cancer agents. These treatments affect many organ systems and can lead to life-threatening toxicities, some of which are dependent on exposure to the drug (Shanholtz, 2001). Knowledge of different molecular tumor characteristics, genetic variants, and drug concentrations could be better utilized to guide and improve dosing (Collins and Varmus, 2015; Groenland et al., 2019), thus reducing morbidity and mortality from both treatment and disease. Cardiovascular disease is another major cause of health care burden globally, accounting for one-third of all deaths in 2015 (Roth et al., 2017). Patients suffering from common cardiovascular disorders such as atrial fibrillation and venous thromboembolism must be treated with anticoagulant therapy, which is associated with serious risks (notably thrombosis if under dosed and bleeding if overdosed). INR-based individualized dosing for warfarin is used routinely, but more recently developed anticoagulants such as apixaban, rivaroxaban, and dabigatran are administered at relatively fixed doses. Benefit of individualized dosing for these newer anticoagulants based on patient-specific factors is possible, but yet to be confirmed.

Infectious disease/antibiotics, cancer/antineoplastic agents, and cardiovascular disorders/anticoagulant therapy are all disease/drug combinations where individualized dosing has been shown to decrease morbidity or mortality. Aminoglycoside regimens that are tailored to each patient with the use of drug concentrations and PK-guided dosing have been shown to shorten length of hospital stay (Destache et al., 1990; Burton et al., 1991; van Lent-Evers et al., 1999) and improve survival rates (Bootman et al., 1979; Whipple et al., 1991) without increasing nephrotoxicity. A number of trials have also been performed that provide evidence for the benefits of individualized dosing in oncology therapy. A study by Evans et al. found that methotrexate dose adjustment based on rate of clearance in leukemia patients resulted in higher rates of remission, specifically in those with B-lineage leukemia (Evans et al., 1998). A more recent study in non-small-cell lung cancer patients found that PK-guided dosing of paclitaxel resulted in reduction of paclitaxel-induced neuropathy (Joerger et al., 2016). PK-guided dosing has also been shown to benefit metastatic colorectal cancer patients taking fluorouracil. In a randomized controlled trial conducted by Gamelin and colleagues, dose individualization of fluorouracil based on PK monitoring improved response rate, reduced serious toxicities, and resulted in a trend toward increased survival (Gamelin et al., 2008). Monitoring is also a method used to facilitate dose individualization with warfarin, which is adjusted based on INR levels. Optimizing the dose of warfarin in individual patients using INR has been shown to improve morbidity and mortality outcomes and is guideline recommended (Veeger et al., 2005; Morgan et al., 2009; Holbrook et al., 2012). Enoxaparin is another anticoagulant that has been proven to benefit from individualized dosing. A prospective study conducted by Barras et al. compared patients on conventional dosing of enoxaparin versus an individualized dosing scheme that took into account both renal function and body composition. The study found lower numbers of bleeding events in patients being treated with enoxaparin in the individualized arm (Barras et al., 2008). Additional research is needed to determine optimal dosing strategies for priority disease/drug combinations.

## Pharmacoeconomics

Pharmacoeconomic methods, including cost-benefit, cost-effectiveness, cost-minimization, and cost-utility analyses play a useful role in allocating limited health care resources (Reeder, 1995). These analyses provide valuable information needed to minimize costs associated with the use of pharmaceutical products. Notably, drug therapy that is not appropriately dosed can lead to significant preventable medical expenses and represents an area of needed improvement. The U.S. cost of nonoptimized drug therapy (drug regimen + adherence) is estimated to be about $528 billion in 2016 or about 16% of health care costs (Watanabe et al., 2018). If drug dosing results in greater efficacy and safety, there should be a significant decrease in health care costs. It is necessary that drug regimens be effectively tailored to each individual in order to optimize use of limited health care resources. One method by which this can be achieved is through the use of TDM to adjust dosing. There are a number of studies showing individualized dosing efforts that have resulted in increased cost-effectiveness, particularly with the use of TDM. Use of TDM to adjust aminoglycoside antibiotic dosing has been shown to decrease mortality (Bootman et al., 1979) and length of hospitalizations, (Burton et al., 1991; van Lent-Evers et al., 1999) resulting in substantial cost savings. Additionally, vancomycin serum concentration monitoring has been shown to reduce nephrotoxicity and subsequently save costs (Mar Fernández de Gatta et al., 1996). TDM has streamlined costs for drugs used to treat epilepsy (Rane et al., 2001), gastrointestinal stromal tumors (Zuidema et al., 2019), and depression (Lundmark et al., 2000; Ostad Haji et al., 2013). TDM could improve cost-effectiveness in many other drug classes as well, such as immunosuppressants, protease inhibitors, and chemotherapeutic agents (Touw et al., 2005).

TDM has also been used to guide treatment decisions for mAbs in the management of inflammatory bowel disease (Crohn’s disease, ulcerative colitis) and rheumatoid arthritis (Mould, 2016). Available evidence does suggest major cost savings with the use of TDM to individualize mAbs directed against tumor necrosis factor (TNF), with no negative impact on effectiveness (Steenholdt et al., 2014; Vande Casteele et al., 2015; Martelli et al., 2017; Guidi et al., 2018). However, the use of biologics (including mAbs) in daily practice continues to be difficult due to the associated expenses. Some techniques used to assess mAbs are not able to be performed at a reasonable cost in daily practice as the costs of the measurements (including transportation costs) are frequently too high (Van Stappen et al., 2016). There are also challenges related to assays and their specificity to individual biologics. Assays assessing adalimumab efficacy measure TNF alpha concentrations as opposed to serum drug concentrations which may present barriers if using these point of care assays for multiple TNF alpha inhibitors (*e.g.,* adalimumab, infliximab, etc.) (Vande Casteele, 2017). With several biologics targeting the cytokine pathway (*e.g.,* tocilizumab, an IL-6 receptor antagonist) (Huang et al., 2005), additional resources will need to be dedicated to validate assay specificity to allow for valid point of care TDM. Precision dosing, *via* TDM or other methods, that optimizes drug therapy and reduces spending will be valuable as health care organizations continue to evolve.

In many countries, the health care system is moving toward value-based payment models, where organizations and providers will be paid based on quality of care and patient health outcomes. Health policy reform is ongoing throughout the world with the goal of lowering health care expenditures and reducing inconsistency in safety and quality of care (Counte et al., 2019). Opportunities to improve the performance of value-based programs by enhancing patient care will be valuable especially for high-cost drugs and diseases. Drugs are a major driver in the increasing cost of health care, in particular biologics and drugs that are critical for health (Califf and Slavitt, 2019). Drugs for cancer, bleeding disorders, infectious diseases, autoimmune disorders, and transplantation account for disproportionately high costs and their demand will continue to rise (Durvasula et al., 2018). Specifically, costly drugs in recent years include tyrosine kinase inhibitors, anti-hepatitis C virus polymerase inhibitors, cystic fibrosis drugs, and mAbs, though many more exist and are frequently unaffordable even in high income countries (Gronde et al., 2017; Hwang et al., 2017). Costly drugs that are used in a value-based or pay-for-performance health care model must lower the total cost of care despite their high price. In order to do so, these drugs must be safe and effective for every patient. If a choice had to be made between two high price drugs, one with a fixed dosing scheme and one with detailed dosing guidance for a variety of patient groups, a value-based system would be more likely to favor the drug that offers more optimal dosing. Regardless of price, precision dosing may provide a commercial advantage when there are multiple drugs in the same class with similar mechanism of action, as efficacy and safety profiles may be improved for drugs with individualized dosing options.

The potential cost savings of individualized dosing approaches have been estimated for different drugs and disease states. For example, personalized dosing of the mAb pembrolizumab could save $0.825 billion annually in the U.S. (Goldstein et al., 2017). This study analyzed the economic impact of the fixed dose of pembrolizumab approved by the FDA versus a weight-based dosing strategy, which had been studied in prior clinical trials with equal efficacy and safety as the fixed dose. In the case of pembrolizumab, the use of weight-based dosing would decrease the amount of drug used in the average patient and avoid unnecessary over dosing. Predictions of cost-effectiveness can also be made before a drug’s approval. Model-based proof of concept analyses such as those performed in eribulin (van Hasselt et al., 2015) and rituximab (Pink et al., 2012) demonstrate the feasibility of model-based approaches to estimating cost-effectiveness early in clinical development. This approach can potentially be applied to assess different doses and identify subgroups of patients who are treated cost-effectively. Another disease area where pharmacoeconomic predictions involving individualized dosing have been made is hemophilia. Patients with severe hemophilia are treated with clotting factor concentrates that prevent arthropathy and other bleeding events. Although prophylaxis with the use of these agents is associated with significant benefit (van den Berg et al., 2001), clotting products such as factor VIII are expensive and therefore not implemented for prophylaxis on a large scale. The therapy is typically dosed by weight, but consideration of other patient characteristics such as age, blood group, and level of von Willebrand factor could help prevent under or overuse of clotting products (McEneny-King et al., 2016). The use of PK methods to individualize factor VIII dosing could also be used improve patient outcomes while improving the cost-effectiveness of therapy (Iannazzo et al., 2017). A Bayesian analysis performed by Björkman supports the use of PK tailoring of factor VIII therapy (Björkman, 2010) to allow dose reductions that would translate to a yearly per-patient savings of $56,000 (McEneny-King et al., 2016). PK-guided prophylaxis has been performed in conjunction with a software application called myPKFit^®^, and results suggest that this approach can result in improved outcomes and optimized factor VIII usage in patients with severe hemophilia without significantly increasing cost of therapy (Mingot-Castellano et al., 2018).

While precision dosing has the opportunity to maximize benefits and savings, barriers exist in practice for cost-effective applications of precise dosing. Precision dosing may require additional costs initially for analysis of drug concentration or other biomarkers that provide information necessary for optimal dose selection. These analyses, though theoretically cost-effective, may require a learning curve for clinicians before expenditures are reduced in clinical practice. Providers may not have the knowledge or experience to adjust their prescribing in response to relevant information, especially if alternate dosing is not on the drug label. Another cost associated with precision dosing is the integration of drug dosing software into EHRs. EHRs have been partially or completely implemented in 99% of U.S hospitals (Pedersen et al., 2017) and are beginning to link to tools that can be used to supply dosing guidance. These clinical decision support tools are being developed to provide patient-specific dose recommendations during the prescription writing process but require a number of validation steps before they can be used. These validation steps are a source of expense but are necessary in order to ensure that the tool will work as expected in patients. More information on precision dosing tool linkage to EHR systems can be found in a review paper by Gonzalez et al. (Gonzalez et al., 2017). Additionally, drug packaging can simultaneously drive up costs and deter precision dosing. For example, many oncology drugs are packaged as single dose vials that do not match up to doses that are administered, leading to wasted leftover drug (Bach et al., 2016). Manufacturers can help address these issues by incorporating individualized dosing recommendations in the prescribing information as well as expanding the availability of drug formulations, volumes, and strengths. More and better formulations that will support weight-based dosing and precision dosing in general are needed. As necessary changes are implemented in the health care system, cost savings are likely to differ widely among drugs, patients, settings, and disease states. Therefore, pharmacoeconomic research will continue to be very useful in determining the value of precision dosing.

## Phase III Trial Patients and Real-World Patient Gap

Implementation of precision dosing requires adequate information and understanding of the dose–exposure and exposure–response relationships across patients in the real-world likely to be administered the drug. When there exists a large difference between the study population evaluated in phase III clinical trials and real-world patients, results from large randomized clinical trials may be difficult to interpret and apply for broad clinical use. All patients deserve an equal opportunity to experience optimal treatment outcome. Should there be concern for enrolling sufficient populations to evaluate absolute benefit–risk of investigational compounds and meet study power requirements, adaptive trial designs offer one potential solution to provide flexibility and improve the efficiency of clinical trials (Pallmann et al., 2018). The anticipated gap between clinical trial patients and real-world patients needs to be identified *a priori* before phase III studies such that evaluation of exposure–response relationships in underrepresented patients may be further investigated in post-marketing studies to inform precision dosing strategies. Though package labeling may indicate dosage adjustments for some patient populations (*e.g.,* elderly, decreased organ function), rarely are the criteria for such determinations explicitly stated. Phase III clinical trials utilize eligibility criteria to establish which patients can participate in the study with consideration to the drug’s mechanism of action, disease characteristics, drug adverse event profile, and the likelihood the clinical trial’s objectives will be met. These inclusion and exclusion criteria ensure the internal validity of clinical trials and minimize risks for vulnerable populations, but often result in the underrepresentation of many real-world patients. Subpopulations, such as pregnant, pediatric, geriatric, intellectually disabled, and physically disabled patients comprise approximately 58% of the U.S. population, yet are often excluded from trial populations (Spong and Bianchi, 2018). While eligibility criteria can protect additional risks of harm in vulnerable patient populations, overly stringent criteria limit the generalizability of clinical trial results to subpopulations.

Exclusion criteria should correlate with the clinical trial’s primary and secondary outcomes and must be well-justified. A systematic review of almost three hundred trials published in high-impact journals between 1994 and 2006 found a majority (84.1%) of trials contained at least one poorly justified exclusion criterion with a quarter of all exclusion criteria being poorly justified in 61.5% of randomized controlled trials (Fowler and Van Spall, 2007). Investigators defined poorly justified criteria as those based on age, race, educational background, socioeconomic status, or other factors with no direct bearing on the condition or intervention. Extensive eligibility criteria may promote a more uniform and homogenous study population but when excluding populations for whom the drug may eventually be used, valuable dose–exposure and exposure–response information cannot be investigated for diverse patient groups. In a recently published draft guidance, the FDA recommends characterizing drug metabolism and clearance across patient populations that may metabolize or clear the drug differently during early drug development to avoid later exclusions in clinical trials (U.S. Food and Drug Administration, 2019). Alternatively, industry sponsors may consider expansion cohorts to allow dose modifications to be used at a reasonably safe dose in specific populations (U.S. Food and Drug Administration, 2019). These recommendations may help include populations for which there are no strong clinical or scientific justifications for exclusion [*e.g.,* those at weight extremes, those with malignancies or certain infections such as human immunodeficiency virus (HIV), children, etc.] but are often excluded on the basis of additional requirements for monitoring and safety precautions. The benefit of including these heterogenous, diverse populations should be carefully assessed as considerably large patient populations will be required to evaluate the potential benefit and risk of investigational drugs. Stringent eligibility criteria lacking strong justification prevent the inclusion and evaluation of the drug’s efficacy and safety in patient subpopulations likely to receive the drug, creating a gap in the applicability of clinical trial results to real-world use.

When evaluating the utility of precision dosing for specific patient populations, the expected scope of the drug usage with regard to patient populations must be determined. For example, if the drug is to be intended for use in pediatric and neonatal patient populations, limitations such as low study consent rates, ethical challenges, limited available blood volume, and lack of robust clinical end points restrict enrollment of these vulnerable subpopulations into clinical trials (Laughon et al., 2014). For these populations lacking phase III clinical data, drug safety and efficacy information are often obtained from post-marketing data sources such as EHRs, registries, and insurance claims data. These post-marketing data sources contain information reflective of real-world drug use patterns and allow for larger and more diverse patient population inclusion with longer follow-up periods, but lack both randomization and consistency in data quality (*e.g.,* coding errors, missing data, and limited validation can be problematic) (**Table 1**). Nevertheless, these data sources can be used to inform different approaches and models to assess the gap between real-world patients and clinical trial patients.

**Table 1:** Examples of post-marketing data used to provide drug information in real-world patient populations and approaches to better characterize and assess the differences between clinical trial and real-world patients.

Post-marketing data	Data type	Advantages	Disadvantages	Examples
Sources (Camm and Fox, 2018)	Claims Data	Encompasses large patient population (10^3^–10^6^); can be used to study rare events and evaluate economic impact	Lack of randomization; data quality concerns (*e.g.,* missing data, coding errors); limited validation; minimal information on health outcomes	Medicare claims data demonstrated decreased risk of ischemic stroke, intracranial hemorrhage and death with dabigatran 150 mg twice daily as compared to warfarin but increased risk of major gastrointestinal hemorrhage in elderly patients with nonvalvular atrial fibrillation. Dabigatran 75 mg twice daily was indistinguishable from warfarin except for a lower risk of intracranial hemorrhage with dabigatran (Graham et al., 2015).
	Registries	Encompasses large and diverse population; captures real time data; can be used to identify cost-effective treatment options	Lack of randomization; data quality concerns (*e.g.,* missing data); data not collected at defined intervals	U.K. transplant registry data suggested significant benefit for graft survival with prolonged-release tacrolimus as compared to immediate-release tacrolimus with a number needed to treat of 14 to avoid one graft loss and 18 to avoid one death (Muduma et al., 2016).
	EHRs	Captures real-time treatment, outcomes and procedures; can be used to study rare conditions	Requires sophisticated data management and statistical tools; data quality concerns (*e.g.,* missing data, coding errors, recall biases); lack of randomization	Electronic health care data were utilized to evaluate the benefits of switching first-line fever coverage from piperacillin-tazobactam to cefepime in pediatric stem cell transplant patients. Researchers saw a reduction in nephrotoxin-associated acute kidney injury episodes with no increases in treatment failures or infection rates (Benoit et al., 2019).
Examples of Approaches and Applications	GIST (ClinicalTrials.gov + EHR data or NHANES data) (Weng et al., 2014; He et al., 2015)	Patient representative analysis of clinical trials using EHR data or public survey datasets (NHANES data); NHANES data not limited to admitted patients and is well-structured and readily analyzed	Univariate model; lack of longitudinal analysis and use of self-reported medical conditions with NHANES data; data quality issues (EHRs and ClinicalTrials.gov carry potential for missing data)	When applied to type II diabetes clinical trials and EHR data, the GIST approach found that most studies are more generalizable with regard to age than they are with regard to hemoglobin A1c (HbA1c). (>70% of studies enroll patients with HbA1c between 7–10.5% though this encompasses only 38% of real-world patients; most studies allow patients age 18–80 years as compared to 10% of the real-world population that falls out of this range) (Weng et al., 2014). He et al. later validated the GIST approach using clinical trial data and NHANES data and concluded patients enrolled in type II diabetes trials are younger, with lower body mass index (BMI) and higher HbA1c than the general patient population (He et al., 2015).
	mGIST (ClinicalTrials.gov + NHANES data) (He et al., 2016)	Patient representative analysis of clinical trials using public survey datasets (NHANES); multivariate model; more effective and efficient in comparing representativeness of multiple study sets; NHANES data not limited to admitted patients and is well-structured and readily analyzed	Lack of longitudinal analysis and use of self-reported medical conditions (NHANES data); does not assess clinical relevance of factors (each variable weighted equally); data quality issues with ClinicalTrials.gov (potential for missing data)	Using the multivariate GIST metric, He et al. concluded that a significant portion of type II diabetic patients are eligible for fewer than 40% of clinical studies. Those aged >70 years are likely not eligible for most studies.
	MAGIC (ClinicalTrials.gov + NHANES data) (He et al., 2016)	Algorithm to identify underrepresented subpopulations in clinical trials; comparable to other methods of characterizing underrepresented population studies; NHANES data not limited to admitted patients and is well-structured and readily analyzed	May yield large number of subgroups with large variable ranges (does not aggregate similar subgroups); similar limitations with data sources as GIST/mGIST (lack of longitudinal analysis, use of self-reported medical conditions, does not assess clinical relevance of factors, data quality issues)	MAGIC identified 50 combinations of underrepresented population subgroups in type II diabetes clinical trials (*e.g.,* elderly obese pre-diabetic female, elderly overweight pre-diabetic male, elderly obese diabetic male, etc.). Researchers also concluded that 94% of type II diabetic patients would qualify for 20% of clinical studies but only a quarter would qualify for half of the studies (He et al., 2016).

EHR, electronic health record; GIST, Generalizability Index for Study Traits; mGIST, Multivariate Generalizability Index for Study Traits; NHANES, National Health and Nutrition Examination Survey; MAGIC, Multivariate Underrepresented Subgroup Identification.

Recently there have been novel approaches proposed to assess and characterize the anticipated gap between clinical trial patients and real-world patients using various sources of post-marketing data. **Table 1** compares the advantages and disadvantages of the sources and applications of post-marketing data. The Generalizability Index for Study Traits (GIST) utilizes information from ClinicalTrials.gov and EHRs to indicate the scale of generalizability to which clinical trial patients reflect real-world patients (Weng et al., 2014). The multiple-trait GIST (mGIST) was subsequently created to expand the utility of GIST to include multiple variables (He et al., 2016). While GIST and mGIST may elucidate differences in clinical trial study populations as compared to real-world patients, identification of underrepresented subgroups can be characterized using Multivariate Underrepresented Subgroup Identification, or MAGIC, which retrieves information from National Health and Nutrition Examination Survey (NHANES) data and ClinicalTrials.gov to identify patient populations not represented in clinical trials (He et al., 2016) (though such identification of appropriate subpopulations assumes there exists a valid comparator control group). In identifying underrepresented subgroups, MAGIC can provide information for future inclusion of these populations in clinical trials. Application of post-marketing data through these approaches can assist in assessing and characterizing the gap between study populations and real-world patients but may be more valuable for suggesting improvements in enrollment diversity for future clinical studies.

The gap between clinical trial patient populations and real-world patient populations presents a major obstacle in precision dosing due to the lack of information available for subpopulations excluded from clinical trials. Although the use of post-marketing data can help provide information to supplement dosing recommendations in subpopulations, including a more diverse patient population in clinical trials by utilizing less stringent inclusion and exclusion criteria can help broaden the applicability of clinical trial results to a larger patient population. Identifying and enrolling these subpopulations will be more reflective of the true target patient population and will be helpful in facilitating precision dosing strategies.

## Conclusion

Precision dosing prioritization can be made by taking into consideration a drug’s therapeutic index, the extent of PK/PD variability, the availability of biomarkers to facilitate individualized dosing, the consequences of imprecise dosing for different disease states, pharmacoeconomics, and differences in dose–exposure and/or exposure–response relationships between phase III trial patients and real-world patients. Each of these variables can be assessed based on their individual and combined impact on efficacy, safety, and cost of drug therapy. By evaluating this information, drugs can be prioritized for precision dosing efforts. There is opportunity for precision dosing framework to become more advanced in the future, and there will be additional factors to examine during drug development and post-approval.

**Figure 2:**
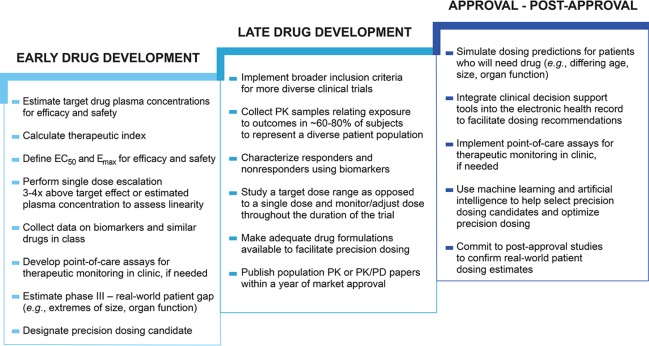
Drug development changes enabling precision dosing. The drug development process approval is generally not designed to facilitate precision dosing. Changes such as studying a target dose range could prime a drug in development for future precision dosing (Maloney, 2017; Peck, 2019), while other changes could facilitate precision dosing in already approved drugs, such as the use of clinical decision support tools to guide dosing. Early drug development encompasses phase I and II clinical trials, late drug development includes phase III clinical trials, and approval – post-approval includes phase IV investigations. *Half maximum effective concentration (EC_50_), Maximum effect (E_max_), pharmacokinetic (PK), pharmacokinetic/pharmacodynamic (PK/PD)*.

For precision dosing to play a larger role in fulfilling public health need, changes are necessary in the drug development process throughout both early development (phase I/II) and late development (phase III) as well as post-approval (phase IV). **Figure 2** suggests actions that could be taken during each stage of drug development to ultimately improve the processes of drug dose selection and optimization in individual patients. Comprehensive information needed to perform precision dosing assessments is not routinely gathered in early drug development. Phase I/II studies should be designed to collect robust data to characterize exposure–response relationships across a wide range of doses. Better understanding of the variability in dose–exposure and exposure–response relationships, as well as disease progression can then be used to inform precision dosing strategies (Peck, 2019). Additional information about similar drugs in class, genomic and nongenomic biomarker data, special populations that will likely use the drug, and the anticipated real-world patient gap are also needed to make an accurate precision dosing assessment. Characterization of the anticipated gap between phase III and real-world patients should be started during early drug development to help assess the potential impact of variability in dose–exposure and exposure–response relationships. This is important to include in precision dosing considerations because the incongruity between study patients and patients who ultimately receive a drug can be a barrier to dose individualization in different populations. For example, if there is little or no phase III trial data in pediatric patients, it may be difficult to determine dosing requirements in this population. In early clinical development it is feasible to estimate the characteristics for patients likely to use the drug once marketed even though the phase III population sample will be much more restricted. This is a good time to begin planning for providing dosing for the market population (*e.g.,* extremes of age, size, organ function, pregnancy, drug interactions). Early development is also when the potential value of feedback-based dosing should be considered. Sufficient information gathered during the early stages of development would allow for better understanding of precision dosing needs for a drug moving into the later stages of drug development.

Once a drug has reached late phase drug development, it should be studied in a population that is generalizable to real-world patients. Many phase III trials have stringent exclusion criteria in order to minimize risk and maximize benefit (*i.e.,* therapeutic efficacy) for the target population, thus increasing the chance for a drug’s approval. The use of a traditional approach to dose selection with a single dose in phase III trials is associated with low success, and is increased when more than one dose is studied and when model-based adaptive designs are used (Looby and Milligan, 2011). If phase III trials were not limited to studying a fixed dose, a wider patient population would likely experience positive outcomes from the drug, though this would also necessitate more robust sample sizes during clinical trials to study drug efficacy and safety across varying doses. Studies conducted using a dose range would allow for a better understanding of the benefit–risk ratio in more types of patients and increase the probability of a drug’s success. Approval of an “optimal dose range” or an “optimal drug plasma concentration exposure range”, rather than the standard one or two doses would permit prescribers to titrate doses within the range based on individual patient characteristics. The optimal drug exposure range for an indication could be identified and subsequently targeted with different doses to achieve optimal exposure across varying patient populations (Maloney, 2017; Neely, 2019). However, in order for this adaptable dosing approach to become a reality, sufficient formulations must be manufactured. Currently, drugs are generally formulated in such a way that makes precision dosing difficult or impossible. Injectable therapies offer great flexibility for dose adjustment, particularly with the use of multi-dose vials which can address the concerns of waste and sterility of single-use vials or ampoules. More commonly used oral formulations, however, pose more barriers for precision dosing. For example, unscored tablets, capsules, and extended release tablets cannot be split and may come in only several strengths that differ by 100% or more. Furthermore, there are hundreds of dosage forms that cannot be crushed or compounded, limiting their use in many patients (Teresk et al., 2017). We likely need more flexible approaches to formulating drugs that require individualization, such as with the use of sachets. However, although this may be more ideal for patients, offering more flexible dosing by diversifying formulation strengths may complicate manufacturing and distribution processes with increased costs. These cost considerations should be evaluated alongside the potential benefit of providing higher quality treatment to a larger group of patients. Other important areas of improvement in drug development include better incorporation of biomarkers and PK sampling into phase III studies. Before drug development is complete, biomarkers should be used to identify responders versus nonresponders and PK samples should be collected to relate exposure to outcomes (Gonzalez et al., 2017). The potential utility of biomarkers to guide dosing should also be characterized and can be done in both early and late stages of drug development. Information gathered during clinical trials such as biomarker assay and PK modeling data that may inform optimal drug dosing is commonly delayed from being published or not published at all, but could be used to develop precision dosing strategies before, after, and at the time of drug approval.

Post-approval studies also play an essential role in the success of precision dosing. After a drug is approved, there continues to be opportunities to refine dosing. The benefit–risk ratio for any given drug is dynamic and can evolve based on new efficacy or safety findings (Curtin and Schulz, 2011). Unfortunately, real-world patient data are underutilized. Not only are patient populations limited during drug development, but multi-year real-world evidence for large numbers of patients are ignored. For example, when a generic drug is approved, the generic’s company routinely adopts the innovator label, disregarding years of real-world experience that if considered could potentially result in individualized or group dosing changes that would improve the overall benefit–risk profile of the drug. The generic company must accept the innovator label or undergo a full re-review, and there is no requirement to update generic dosing recommendations to reflect real-world patient experience. Regulatory incentives and requirements should be implemented to encourage the update of dosing recommendations based on real-world data. Fortunately, the importance of updating generic drug labels to prevent the dissemination of out-of-date drug information has been recognized (Gingery, 2018; Fromer, 2019).

There should also be incentives for the development of precision dosing infrastructure essential to the facilitation of dosing recommendations such as point of care assays for therapeutic monitoring and clinical decision support tools. In drug development, highly sensitive and specific assays are required to accurately detect lower levels of quantitation and establish therapeutic concentrations (Unger et al., 2013; U.S. Food and Drug Administration, 2018). Once therapeutic concentrations have been established, assays to be used for commercial and/or clinical use need to be reliable and specific at therapeutic concentrations (Kang and Lee, 2009). In addition, the accuracy, precision, sensitivity, and specificity of the assay should be documented and assessed on a routine basis (Gross, 2002). During research and development, the turnaround time for assay data will be more prolonged (months), but if used in practice, data on drug concentrations should be rapidly available within days to clinicians. For some drugs it may be reasonable to initiate the drug and adjust the dose later if necessary based on a lab or biomarker. These assays and tools would ideally be available at the time of approval and have the opportunity to make a large impact on patient care, especially as the use of EHRs becomes more prevalent. It is important to note that assay variability may be greater than that of the therapeutic range, which presents an additional challenge to consider when attempting to adjust dosing based on assays. Additional considerations include how long it takes a drug to impact a biomarker and the timing of measurement, depending on the biomarker that is being measured because these can impact the results of the assay. Other innovative technologies such as machine learning and artificial intelligence may also be used to help select precision dosing candidates and optimize dosing in the future. These technologies may be used to find clinically meaningful patterns in large amounts of data (Shah et al., 2019), which can then be used to inform drug dosing.

Precision dosing has the potential to transform health care by maximizing benefits while minimizing risks involved in drug therapy. While the impact of precision dosing is likely to be substantial for some drugs, it may not be necessary or feasible to implement for every drug or drug class. Therefore, identifying the factors that make drugs good targets for precision dosing will help direct resources to where they will be most useful. The selection of high priority drugs and drug classes for precision dosing could be key in moving toward better efficacy, safety, and cost-effectiveness in health care.

## Author Contributions

RT and CP wrote the manuscript with guidance from DG. JRP, JHP, DW, and PW reviewed the manuscript critically. All authors approved the final version of the manuscript to be published.

## Funding

RT is supported by the National Institute of General Medical Sciences of the National Institutes of Health under Award Number T32GM086330. CP is supported by United Therapeutics for post-doctoral fellowship. DG receives support for research from the *Eunice Kennedy Shriver* National Institute of Child Health and Human Development (NICHD, 5K23HD083465 and 5R01HD096435). In addition, the authors would like to acknowledge the generous research support provided by the Eshelman Institute for Innovation (EII) at the UNC Eshelman School of Pharmacy. The content is solely the responsibility of the authors and does not necessarily represent the official views of National Institutes of Health.

## Conflict of Interest

JHP declares consulting and research support from Novartis and research support from Amgen, Merck. and Boehringer Ingelheim. DW serves on the Simulations Plus Board of Directors. PW consults widely within the pharmaceutical industry. DG received a travel grant through University of North Carolina at Chapel Hill to give a presentation at Boehringer Ingelheim.

The remaining authors declare that the research was conducted in the absence of any commercial or financial relationships that could be construed as a potential conflict of interest.
